# Comparison of cross-platform technologies for EGFR T790M testing in patients with non-small cell lung cancer

**DOI:** 10.18632/oncotarget.19007

**Published:** 2017-07-05

**Authors:** Xuefei Li, Caicun Zhou

**Affiliations:** ^1^ Department of Lung Cancer and Immunology, Shanghai Pulmonary Hospital, Tongji University School of Medicine, Pulmonary Cancer institute, Tongji University School of Medicine, Shanghai, P. R. China; ^2^ Department of Medical Oncology, Shanghai Pulmonary Hospital, Tongji University, Tongji University Medical School Cancer Institute, Shanghai, P. R. China

**Keywords:** EGFR mutations, TKI resistance, circulating tumor DNA, NSCLC, companion diagnostics

## Abstract

Somatic mutations in the gene encoding epidermal growth factor receptor (EGFR) play an important role in determining targeted treatment modalities in non-small cell lung cancer (NSCLC). The EGFR T790M mutation emerges in approximately 50% of cases who acquire resistance to tyrosine kinase inhibitors. Detecting EGFR T790M mutation in tumor tissue is challenging due to heterogeneity of the tumor, low abundance of the mutation and difficulty for re-biopsy in patients with advanced disease. Alternatively, circulating tumor DNA (ctDNA) has been proposed as a non-invasive method for mutational analysis. The presence of EGFR mutations in ctDNA predicts response to the EGFR TKIs in the first-line setting. Molecular testing is now considered a standard care for NSCLC. The advent of standard commercially available kits and targeted mutational analysis has revolutionized the accuracy of mutation detection platforms for detection of EGFR mutations. Our review provides an overview of various commonly used platforms for detecting EGFR T790M mutation in tumor tissue and plasma.

## INTRODUCTION

Lung cancer is a major cause of cancer deaths with approximately 80% of cases accounting to non-small cell lung cancer (NSCLC) [1]. In NSCLC target therapy, epidermal growth factor receptor (EGFR) is a promising candidate [2]. The frequency of EGFR mutation among Asian NSCLC populations is approximately 30% compared with approximately 10% in Caucasians [3-5]. EGFR TKIs like gefitinib, erlotinib, and afatinib are used for EGFR targeted therapy in NSCLC [6, 7]. The mode of action of tyrosine kinase inhibitors is to inhibit the kinase activation and signal transduction downstream by binding to the ATP binding site of the kinase domain of EGFR [7]. This targeted therapy has shown 56 to 74% of response rate with median of 10-14 months of progression free survival (PFS) [8, 9].

Most common mutations of EGFR gene include in-frame deletions of exon 19 and heterozygous mutations of exon 21 [7]. The correlation between EGFR mutations and EGFR TKI sensitivity has shown prognostic potential as demonstrated from various clinical trials [10, 11]. Although, patients respond well, initially to EGFR TKIs, majority of them acquire resistance due to the emergence of secondary T790M resistance mutation which abrogates the TKIs inhibitory action [12-15]. This can be overcome by use of second-generation EGFR inhibitors (afatinib and dacomitinib), however, these inhibitors showed low response rate ( < 10%) and low PFS ( < 4 months) [16-18]. They are also associated with skin and gastrointestinal toxic effects [19, 20]. A third-generation EGFR TKI that is potent to T790M resistance mutation is AZD9291. This is shown to be effective with a response rate of 61% and limited skin and gastrointestinal adverse events in patients who developed T790M mediated resistance to EGFR TKIs. AZD9291 also targets EGFR sensitizing mutations (exon 19 deletion and L858R) [21, 22].

Monitoring post-TKI progression events in tumor tissue has drawn much importance as it assists in designing therapeutic strategies to overcome resistant mechanisms. In order to study these mechanisms of resistance re-biopsies are recommended, however in clinical practice this becomes challenging due to invasive procedure and heterogeneity of the tumor tissue [23, 24]. A non-invasive alternative to tissue is circulating tumor DNA (ctDNA) that has emerged recently and is reported as specific and sensitive biomarker for EGFR mutation detection. Mutations detected in tumor tissue showed high concordance with those observed in plasma ctDNA [25-27].

Several clinical platforms are available to detect EGFR mutations including amplification refractory mutation system (ARMS), cobas TaqMan-based PCR, digital polymerase chain reaction (PCR) including droplet digital PCR (ddPCR) and BEAMing (beads, emulsions, amplification, and magnetics) digital PCR, mutant-enriched PCR, high-resolution melting (HRM) analysis, denaturing high performance liquid chromatography (DHPLC) and next generation sequencing (NGS). These techniques vary in their sensitivity and their specificity in their rate of detection in plasma and tumor tissue.

Real-time monitoring of EGFR mutations is essential for determining appropriate treatment strategies; therefore, less invasive procedures combined with highly sensitivity, specificity, cost-effective diagnostic platform remains an unmet need. Hence, we review the existing EGFR T790M mutation testing technologies and their sensitivity and specificity in detecting these mutations in plasma, tissue and bodily fluid samples.

## COMPANION DIAGNOSTIC PLATFORMS FOR EGFR T790M MUTATION DETECTION

Currently several PCR based diagnostic platforms are available for EGFR mutation detection including cobas, ARMS, BEAMing, droplet PCR, HRM, DHPLC, mass spectrometry genotyping, electric field-induced release and measurement (EFIRM) and NGS. Here we review the varying sensitivity and specificity of most widely used platforms and their use in plasma and tumor tissue. Table 1 represents the salient features of the companion diagnostic platforms used for EGFR mutation detection.

**Table 1:** Companion diagnostic platforms for EGFR mutation detection

Platform	Cobas	ARMS		Digital PCR			NGS	
Commercially available kit/brand	Roche [28]	Qiagen [32,33]	Amoydx [34]	Bio-rad ddPCR [37]	Sysmex Inostics BEAMing Digital PCR [36]	Thermo^™^ QuantStudio 3D Digital PCR System [38]	Illumina Miseq [39]	Thermo Fisher Ion Torrent [41]
Technique	Real-time PCR using TaqMan Probes	ARMS Scorpion primers with PCR technology	ARMS PCR based technology with florescent probe	Water-emulsion droplet technology	Emulsion PCR with magnetic beads and flow cytometry	Chip based technology	Sequencing by synthesis technology	Semiconductor chip based technology
EGFR Mutations coverage	42 mutations in exon 18,19,20 and 21 of EGFR gene	29 mutations in exon 18,19,20 and 21 of EGFR gene	29 mutations in exon 18,19,20 and 21 of EGFR gene	Broad mutation coverage requires specific primer/probe design				
Turnaround time	1 day	<1 day	<1 day	<1 day	7∼10 days	<1 day	8∼10 days	8∼10 days
Characteristics	Qualitative and semi-quantitative	Qualitative	Qualitative	Quantitative	Quantitative	Quantitative	Quantitative	Quantitative
Effort	Less laborious	Less laborious	Less laborious	Less laborious	Intermediate	Less laborious	High	High
Analysis of results	Simple, Automated detection through cobas z 480 analyzer.	Simple	Simple	Intermediate, Quantasoft software measures the positive and negative droplets and gives output in copies/µl of the target DNA.	Intermediate	Intermediate	Complicate	Complicate, Automated analysis through Ion Reporter
Sensitivity	2∼3% for FFPET, 100copies/ml for plasma (T790M)	1%	1% for FFPET, 0.2% for plasma SuperARMS)	0.2%	0.01%	0.1%	0.1%∼0.5%	0.1%∼0.5%
Advantages	Tissue and Plasma samples can be run on the same plate. FDA approved method for mutational analysis.	Low Complexity. FDA approved method for mutational analysis.	Low Complexity. CFDA approved method for mutational analysis.	Absolute quantification, high sensitivity and specificity			1. High throughput; 2. Can read the repetitive sequence	1. Input as less as 1ng gDNA. 2.. Low cost;
Disadvantages	Does not give absolute quantification of the mutation. Detects only known mutations.			Detects only known targeted mutations			1.Longer turnaround time 2. High cost (fluorescence); 3. Complicate library preparation.	1.Longer turnaround time 2. Low throughput; 3. Complicate library preparation.

### Cobas (Roche)

**Figure 1:**
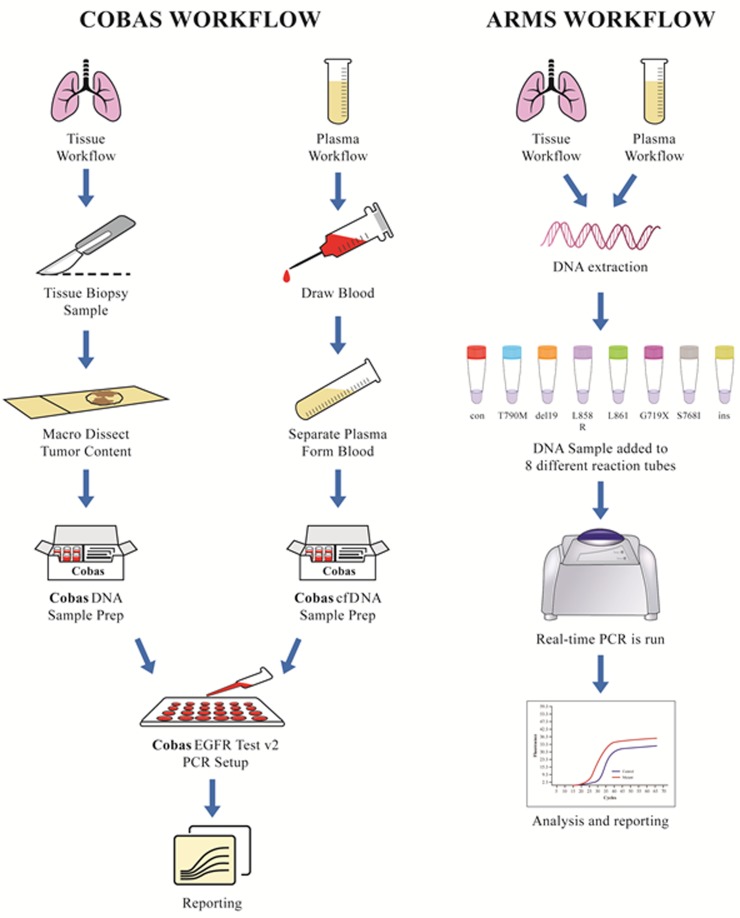
Workflow of cobas (Roche) [28] and ARMS (Qiagen) [29] The workflow includes sample collection, isolation of DNA from the sample using specific DNA sample preparation kit, running the sample DNA in real-time PCR and results are used for clinical interpretation and targeted therapy

This is a real-time PCR based technique that identifies 42 locus mutations of EGFR including T790M. The procedure has two steps, step one is extraction of DNA from tissue or plasma and the second step is amplification of DNA using specific primers and detection using probes with fluorescent dyes. It is designed to run both tissue and plasma samples on the same plate thus giving clinicians the ease of comparison for planning therapeutic strategies. Plasma samples are processed using **cobas** cfDNA sample preparation kit after separating plasma from the whole blood whereas, for tissue samples **cobas** DNA sample preparation kit is used for extraction of DNA. After sample preparation, amplification and detection is done by running the samples together on the same plate in PCR , thus providing a head to head comparison of tissue with plasma [28]. Figure 1 depicts the workflow of cobas in tissue and plasma.

## ARMS

Allele specific polymerase chain reaction is designed using sequence specific PCR primers and is useful in detecting small deletions or single base mutations [30]. Specific mutated sequences are amplified selectively as Taq DNA polymerase distinguishes a match and a mismatch at 3’ end of the primer, thus amplifying only the target allele DNA. When there is full match good amplification occurs and in mismatch low background amplification is observed. PCR primers covalently bond to a probe; fluorophore of the probe interacts with a quencher (incorporated in the probe) reducing fluorescence. During PCR the probe binds to the amplicon separating the fluorophore and the quencher thus increasing fluorescence in the PCR tube [31].

**Figure 2:**
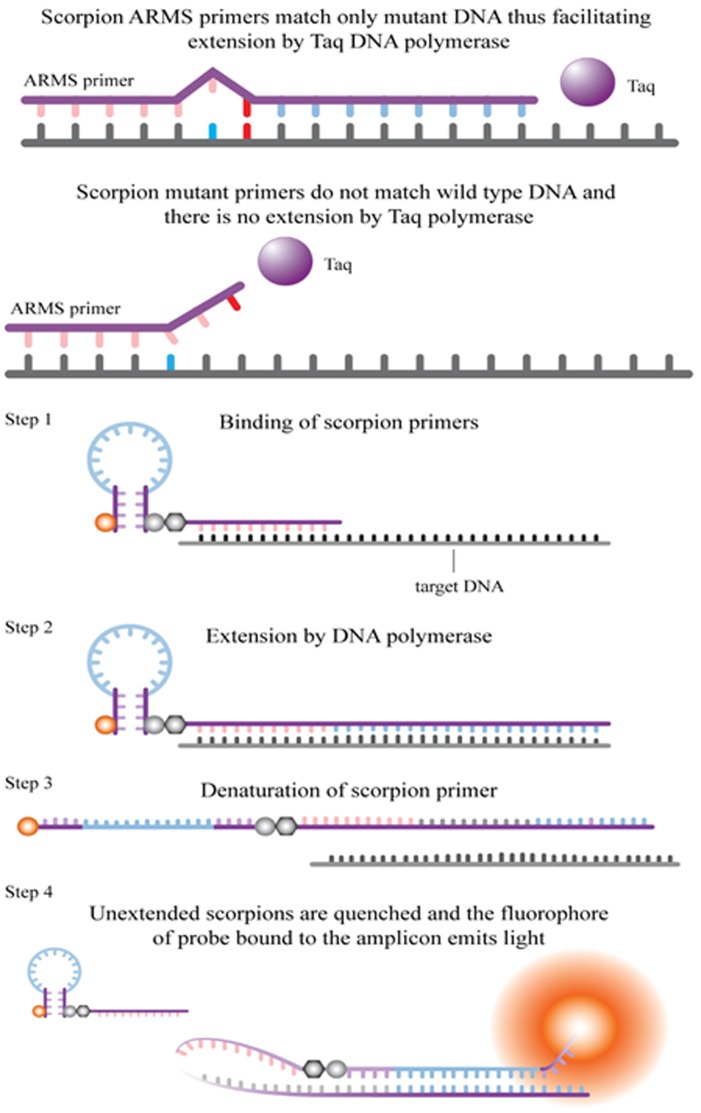
Principle of ARMS (Qiagen) [29]

ARMS (Qiagen): EGFR RGQ PCR Kit version 2 is a diagnostic kit that detects mutations using real-time PCR on the Rotor-Gene Q 5plex HRM instrument. The procedure has two steps, first step consists of the control assay for assessing the total sample DNA and second step has both control and mutation assay to assess mutated DNA [32, 33]. Figure 2 depicts the principle of ARMS.

ARMS (AmoyDx): AmoyDx^®^ EGFR Mutation Detection Test (CE-IVD) is a diagnostic kit that detects EGFR mutations in exon 18, 19, 20 and 21. This technology works using two step PCR amplification procedures combined with novel fluorescent probe design and can be used for fresh or frozen tissue samples, blood serum or plasma [34].

### Digital PCR

Digital PCR clonally amplifies and quantifies nucleic acids. It can amplify and generate amplicons derived from one template using very less sample. Different alleles can be distinguished using fluorophores or sequencing. It is superior to conventional PCR as it transforms the exponential analog signals and gives a linear digital signal output suitable for statistical analysis [35].

**Figure 3:**
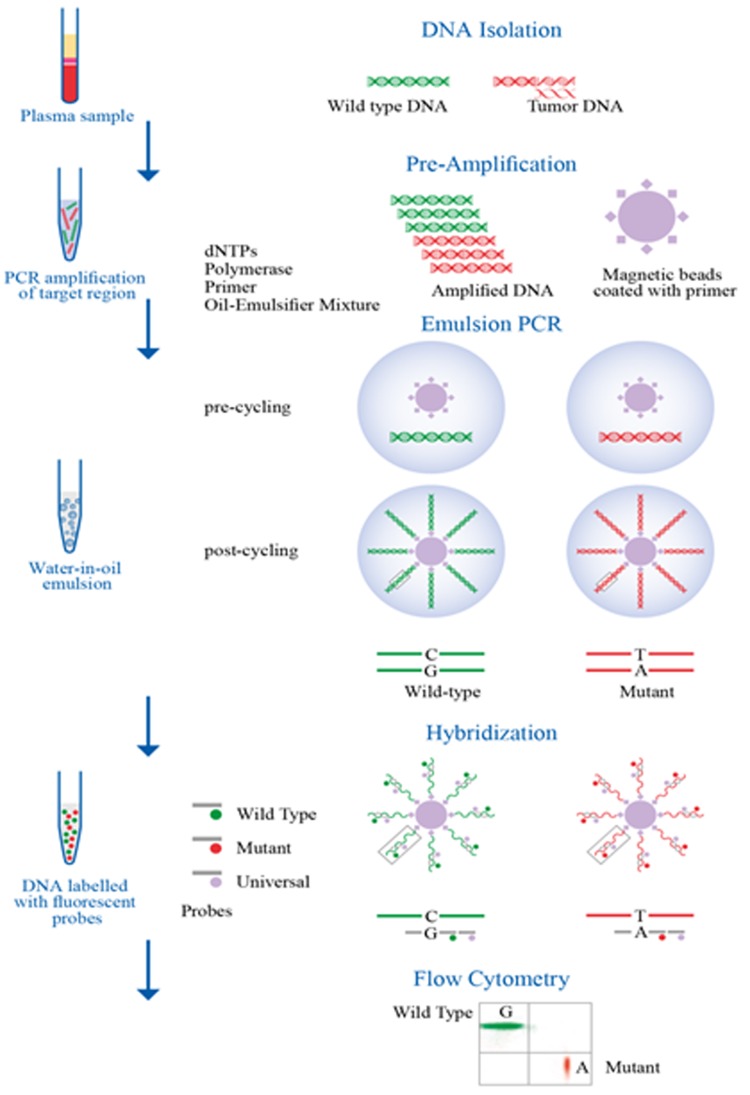
Work flow of Digital PCR (BEAMing) [36] Droplets are generated using droplet generator and are read using droplet reader. However, QuantStudio digital PCR has much simpler workflow which makes use of chip based technology, the sample is loaded and PCR amplified and the results are read and analyzed using system based software.

Sysmex Inostics BEAMing Digital PCR technology is a highly sensitive platform that combines emulsion PCR with magnetic beads and flow cytometry. The workflow involves isolation of DNA and amplification of DNA by PCR. The process involves transformation of a population of DNA molecules into a population of beads coated with primers. This is followed by emulsion PCR and the DNA is hybridised with fluorescent probes. Flow cytometry is performed to read the results [36]. Figure 3 represents the workflow of BEAMing digital PCR.

**Figure 4:**
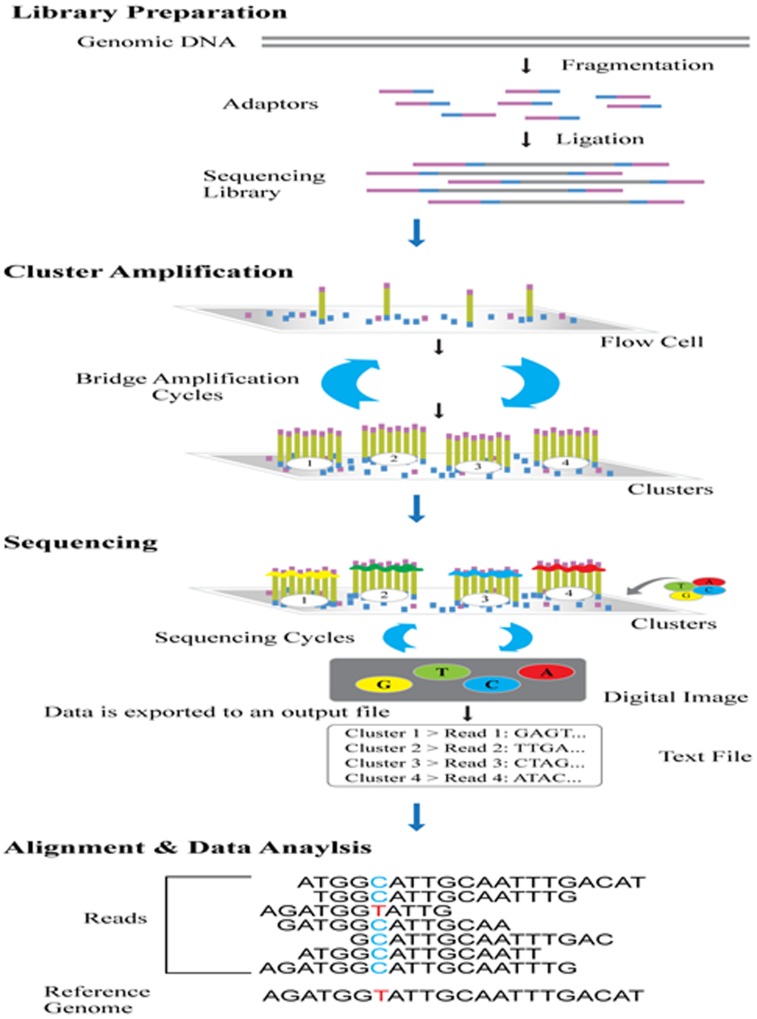
Work flow of NGS (Ilumina) [39] The methodology comprises of template preparation, sequencing, imaging and analysis. The workflow involves library preparation, cluster generations, sequencing, alignment and data analysis. Genomic DNA is fragmented and ligated using 5’ and 3’ adapter ligation to prepare NGS library. These fragments are amplified by PCR and gel purified. They are loaded into a flow cell and hybridisation takes place. Through bridge amplification the bound fragments are amplified into a clonal cluster. These are then sequenced base-by-base using reversible terminator based method thus eliminating sequence context specific errors. After sequencing bioinformatics software is used to align the resultant reads to reference genome thus identifying the differences.

Droplet Digital PCR Bio-rad technology is based on the water-emulsion droplet technology. DNA sample containing the target DNA is fractionated into 20,000 droplets. End-point PCR amplifies each droplet containing target DNA. Quantification of target DNA is done by counting the positive droplets. This method provides the absolute and precise count of target DNA without the standard curves and has higher sensitivity than real-time PCR [37].

QuantStudio 3D Digital PCR uses a sealed chip technology. It is affordable and has 50% less price compared to other platforms. The workflow involves diluting the control DNA, digital PCR reaction is run after mixing control DNA, master mix and reference assays. PCR reaction is loaded onto a QuantStudio^®^ 3D Digital PCR 20K chip, lid is applied and loaded with immersion fluid and sealed. The chip is thermal cycled and the results are read and analyzed using QuantStudio™ 3D Digital PCR Instrument [38].

### NGS

Next-generation sequencing has revolutionized biological research in genome analysis. Illumina MiSeq System is used for targeted genome sequencing and MiSeqDx System is used in molecular diagnostics [39]. Miseq performs sequencing by synthesis technology, a reversible terminator-based method that detects single bases while incorporation into the DNA strands, producing exceptional data quality. This base by base sequencing eliminates errors and produces high quality results. It has simple work flow and has genomic analysis platforms for data analysis and sharing [40]. Thermofisher Ion Torrent NGS technology is powered by semiconductor chips and is simple, scalable and cost-effective method used for targeted sequencing. Ion AmpliSeq technology can amplify thousands of targets using 1ng of genomic DNA or RNA. It can be used for formalin fixed paraffin embedded (FFPE) samples or ctDNA. Sequencing workflow takes less than 2 days. Ion Torrent Oncomine cfDNA Assays can detect mutations at level of 0.1% in genes. Oncomine Lung cfDNA Assay can detect several hotspots in EGFR genes including T790M [41-43].

## SAMPLES FOR EGFR T790M DETECTION

Tumor biopsy is traditionally used for obtaining information on diagnosis, prognosis, recurrence, drug response and drug resistance. With the advent of targeted therapy, it is now important to continuously monitor the molecular alterations emerging in the tissue which demands a repeat biopsy. Obtaining serial repeat biopsies for real-time monitoring of the disease becomes challenging due to the invasiveness, impractical accessibility, and heterogeneity of tumor tissue [23, 24].

Alternatively, plasma derived ctDNA is promising due to its minimal invasive extraction that could facilitate the monitoring of EGFR mutations [1, 44, 45]. Several studies have indicated that ctDNA is likely to derive from tumor lesions and metastatic sites, possibly representing the patients tumor genome [46, 47]. Plasma ctDNA is promising for mutation detection due to the ease of accessibility, convenience and practicality [27]. It has potential in monitoring the real time disease burden and progression by characterizing intra-tumor and inter-tumor heterogeneity [48, 49].

Studies on use of other bodily fluids in mutation detection in lung cancer are limited. Saliva, urine and pleural effusions are some of the clinically available bio-samples that are potentially used in EGFR mutation testing. A core technology called EFIRM has been used for EGFR mutations detection using saliva. Good correlation was observed in EGFR mutation detection between EFIRM and cobas [50]. Several studies have shown use of malignant pleural effusions as an alternative for tissue and blood using PCR for EGFR mutation detection and monitoring [51-53]. Urinary ctDNA has emerged as completely non-invasive sample for assessing disease progression and treatment response in T790M resistant mutation patients. Most studies have used PCR based technology alone or in combination with NGS [54-57]. A study on kinetics of monitoring T790M mutation in urinary samples revealed 68% of patients with T790M mutation post-TKI treatment using PCR coupled with MiSeq. Among these positive patients 10 had similar results with tissue biopsy, three patients who were negative in tissue were detected to be positive in plasma and urine [55]. Another study reported 72% concordance between urine and tissue results for detecting T790M mutation. Plasma and urine detected additional T790M positive cases that were missed by tissue biopsy [58].

## COMPARISON OF DIAGNOSTIC PLATFORMS IN EGFR T790M DETECTION IN PLASMA AND TISSUE

Molecular testing for EGFR gene alterations is considered a standard of care in NSCLC patients. Various treatments guidelines from American Society for Clinical Oncology (ASCO), College of American Pathologists (CAP), International Association for the Study of Lung Cancer (IASLC), Association for Molecular Pathology, and the US National Comprehensive Cancer Network support genetic mutation testing for treatment modalities [59-61]. The guidelines for molecular testing of EGFR mutations recommend a validated mutation method with sufficient performance characteristics with turnaround time of 2 weeks and in case of secondary or acquired resistance to TKIs the method should be sensitive enough to detect secondary mutation (T790M) [60]. Even the new European guidelines encourage coverage of exons 18-21 for mutation detection in NSCLC pateints [62]. United states FDA approved cobas (Roche) as a companion diagnostic tool for EGFR mutations detection (exon 19 deletions, L858R in exon 21 and exon 20 insertions including T790M) using tissue or plasma for TKI targeted therapies (erlotinib and osimertinib) and ARMS therascreen (Qiagen) as companion diagnostics for detecting exon 19 deletions and exon 21 (L858R) substitution mutations using tissue for afatinib selection [63].

In identifying EGFR mutations, concordance between tissue and plasma plays an important role to address the issue of liquid biopsies to serve as molecular substitute for tissue. Studies have reported 100% specificity and sensitivity of ctDNA with concordance rate ranging from 27.5%-100% between ctDNA and tissue biopsy for various EGFR mutations [64-69]. A phase IV, open-label, single-arm study in Caucasian NSCLC patients (*N* = 652) demonstrated 94% concordance for EGFR mutations detected (by ARMS, Qiagen) between plasma and tumor tissue in a study evaluating efficacy and safety of gefitinib [26]. In a cross platform comparison study, the concordance for T790M mutation between plasma and ctDNA was 57%, 48%, 74% and 70% using cobas (Roche), ARMS (Qiagen), ddPCR (Bio-rad) and BEAMing dPCR, respectively between plasma ctDNA and tissue in Chinese NSCLC patients. The digital platforms outperformed to the non-digital ones in sensitivity and concordance in T790M mutation detection [70]. Additional studies on concordance of EGFR T790M mutation detection in tumor and plasma are summarized in Table 2. These studies report wide range of concordance range 48-94%, sensitivities (29-81.8%) and specificities (83-100%). This variability in concordance, sensitivities and specificities are heavily technology driven.

**Table 2:** Concordance of EGFR T790M mutation detection in tumor and plasma

S.No	Method		Sample	Parameters			Study group
	Plasma detection	Tissue detection		Sensitivity	Specificity	Concordance with tissue	
1	Cobas (Roche)	Cobas (Roche)	Plasma *N* = 38	41%	100%	57%	Thress *et al.* [70]
	ddPCR (Bio-rad)			71%	83%	74%	
	BEAMing			71%	67%	70%	
	ARMS Qiagen			29%	100%	48%	
2	Cobas (Roche)	Cobas (Roche)	Plasma *N* = 153	64%	98%	86%	Karlovich C *et al.* [98]
	BEAMing			73%	50%	67%	
3	BEAMing (Sysmex)	Cobas (Roche)	Plasma *N* = 216	70.3%	69.0%	NR	Oxnard GR *et al.* [115]
4	ddPCR (Bio-rad)	ARMS (AmoyDx)	Plasma *N* = 117	81.25%	100%	81.25%	Zheng *et al.* [91]
5	ddPCR (Bio-rad)	ddPCR (Biorad)	Plasma *N* = 18	81.8%	85.7%	83.3%	Ishii H *et al.* [90]
6	ddPCR (Bio-Rad)	ddPCR (Biorad)	Plasma *N* = 41	64.5%	70.0%	65.9%	Takahama T *et al.* [116]
7	Picoliter-ddPCR (RainDance)	ARMS (Qiagen)	Plasma *N* = 10	71%	NR	80%	Seki *et al.* [117]
8	NGS (Illumina, MiSeq)	Cobas (Roche) and ARMS (Qiagen)	Plasma *N* = 60	93%	94%	NR	Reckamp KL *et al.* [58]
9	PANAMutyper R EGFR kit	Ion Torrent NGS	Plasma *N* = 39	58%	68%	63%	Han J Y *et al.* [118]
10	cSMART	ARMS (AmoyDx)	Plasma *N* = 61	100%	NR	98.4%	Chai X *et al.* [119]
11	NGS (MiSeq)	PCR/FISH/NGS (MiSeq)	Plasma *N* = 15	81.8%	100%	86%	Paweletz *et al.* [95]

Several studies have demonstrated use of various platforms for EGFR T790M detection both in plasma (Table 3) and tissue samples (Table 4). Direct sequencing is widely used in EGFR mutation detection. Studies have reported detection limit of direct sequencing to be around 25-30%. This method is complex, time consuming and not standardized in terms of clinical laboratory practice [71-73]. Although, direct sequencing has drawbacks with low sensitivity, several studies have reported use of direct sequencing in detecting EGFR T790M with detection rate ranging from 0-50%. This disparity could be attributed to the low abundance of T790M mutation (due to less sensitivity of the technique mutation is not detected) and also to small sample size (instances where higher detection rates are reported) [71, 74-81]. Some studies compared direct sequencing with other techniques (mutant-enriched PCR, RFLP-PCR, LNA-PCR, Mutation-biased PCR) in T790M mutation detection and demonstrated higher detection rates by other sensitive methods [74, 76-78, 80].

**Table 3:** Comparison of EGFR T790M detection platforms in plasma

S.No	Method	Sample	EGFR T790M detection rate %		Study Group
			Treatment Naive/Pre-TKI	Post-TKI	
1	BEAMing	Plasma *N* = 44	4.8	43.5	Taniguchi *et al.* [106]
2	Scorpion ARMS	Plasma *N* = 26	34.8	64	Maheswaran *et al.* [109]
3	ARMS	Plasma *N* = 135	5.8	31.1	Wang Z *et al.* [89]
	Digital PCR		25.2	43.0	
4	Mutant-enriched PCR	Plasma *N* = 33	NA	36.4	He *et al.* [74]
	Direct Sequencing		NA	6.1	
5	Cobas (Roche)	Plasma *N* = 23	0	39	Sorensen *et al.* [99]
6	ddPCR	Plasma *N* = 49	-	28.6	Lee *et al.* [104]
7	SABER	Plasma *N* = 75	-	28	Sakai *et al.* [120]
8	ddPCR	Plasma *N* = 12	-	41.7	Isobe K *et al.* [92]
9	Mutation-biased PCR	Plasma *N* = 58	-	40	Sueoka-Aragane N *et al.* [112]
10	Mutation-biased PCR	Plasma *N* = 19	-	53	Nakamura T *et al.* [78]
	PNA-LNA PCR		-	15.7	
	Cycleave PCR		-	26.3	
	ASO-PCR		-	31.5	
	Direct sequencing		-	31.5	
11	Cobas (Roche)	Plasma *N* = 15	0	33.3	Marchetti A *et al.* [100]
	NGS (Roche)		0	33.3	
12	Cobas (Roche)	Plasma *N* = 238	0.8	2.01	Mok T *et al.* [88]
13	NGS (Illumina) Hi Seq	Plasma *N* = 45	-	42.2	Jin Y et al. [114]
14	NGS (MiSeq)	Plasma *N* = 15	-	60	Paweletz *et al.* [95]
15	Ion Torrent PGM NGS	Plasma *N* = 190		16.8	Uchida J *et al.* [121]

‘-‘ :Not reported.

**Table 4:** Comparison of EGFR T790M detection platforms in tissue

S.No	Method	Sample	EGFR T790M Detection rate %		Study group
			Treatment Naive/Pre-treatment	Post-TKI	
1	Scorpion ARMS	Tissue *N* = 29	0	48.3	Chen HJ *et al.* [84]
2	Direct sequencing	Tissue *N* = 14	0	50	Kosaka *et al.* [75]
3	ARMS	Tissue *N* = 10	-	0	Zhang *et al.* [85]
	ddPCR		-	50	
4	Standard HRM	Tissue *N* = 146	0	-	Hashida *et al.* [107]
	MEC-HRM		13	-	
5	SABER	Tissue *N* = 28	7	-	Sakai *et al.*[120]
6	Ion Torrent PGM NGS	Tissue *N* = 15	-	60	Masago *et al.* [94]
7	ddPCR	Tissue *N* = 12	83.3	-	Isobe K *et al.* [92]
8	MALDI-TOF MS	Tissue *N* = 54	7.1	-	Su K.Y *et al.* [97]
	NGS		14.3	-	
9	PNA-clamping PCR	Tissue *N* = 50	-	68	Costa C *et al.* [110]
10	ddPCR	Tissue *N* = 78	6.4	-	Xu *et al.* [93]
11	ACB-ARMS PCR	Tissue *N* = 27	22.2	-	Zhao J *et al.* [83]
12	PNA-clamping PCR	Tissue *N* = 147	8.2	-	Oh *et al.* [76]
	Direct sequencing		0	-	
13	ddPCR	Tissue *N* = 373	79.9	-	Watanabe M *et al.*[105]
14	Direct sequencing	Tissue + other clinical samples *N* = 280	0.3	1.05	Inukai M *et al.* [77]
	Mutant-enriched PCR		3.5	3.1	
15	TaqMan PCR	Tissue *N* = 129	35	-	Rosell R *et al.* [122]
16	SARMS	Tissue *N* = 38	0	-	Fujita Y *et al.* [86]
	Colony hybridisation		79	-	
17	Direct sequencing	Tissue *N* = 98	2	-	Sequist LV *et al.* [71]
18	Direct sequencing	Tissue+other clinical samples *N* = 1261	0.5	-	Wu JY *et al.* [79]
19	NGS (Miseq/Hiseq2000/Hiseq2500)	Tissue *N* = 209	0.48	-	Hagemann IS *et al.*[108]
20	LNA-PCR sequencing	Tissue *N* = 155	-	62	Yu HA *et al.* [111]
21	Direct sequencing	Tissue+other clinical samples *N* = 69	-	49	Arcila ME *et al.* [80]
	RFLP-PCR	Tissue+other clinical samples *N* = 45	-	53	
	LNA-PCR sequencing	Tissue+other clinical samples *N* = 64	-	70	
22	TaqMan PCR	Tissue+other clinical samples *N* = 15	-	40	Molina-Vila MA *et al.* [123]
23	AMRS	Tissue *N* = 609	0.8	-	Mok TS *et al.* [87]
24	Direct sequencing	Tissue *N* = 74	-	1.35	Soh J *et al.* [81]
25	Cobas(Roche)/ARMS (Qiagen)	Tissue *N* = 148	-	53	Sequist LV *et al.* [101]
26	Cobas (Roche)	Tissue *N* = 222	-	62	Janne PA *et al.* [21]
27	ARMS	Tissue *N* = 134	6.8	28.4	Yu J *et al.* [124]
28	NGS (MiSeq)	Tissue = 15	-	73.3	Paweletz *et al.* [95]
29	NGS (AmpliSeq cancer hotspot panel v2)	Tissue N = 43	-	79	Belchis DA *et al.* [96]

‘-‘: not reported

ARMS is another most commonly used method for EGFR mutation testing both in plasma and tissue [26, 70,76-78, 82-88]. Though it produces good specificity, it lacks sensitivity when compared to HRM, ddPCR, cobas, colony hybridization and BEAMing [70, 83, 85, 86, 89]. Another study used a method combining allele-specific competitive blocker (ACB) with TaqMan quantitative PCR ARMS called ACB-ARMS PCR for EGFR T790M testing and found 22.2% T790M mutation detection rate as compared to scorpion ARMS (0.0%) in tissue samples [83].

Quantification platforms like ddPCR and NGS are also widely used in T790M mutation detection especially in dynamic monitoring during TKI therapy. Ishii *et al.* reported high sensitivity (82%) and specificity (86%) of digital PCR (bio-rad) in detecting T790M mutation using plasma ctDNA with concordance of 83.3% with tumor tissue. Qualitatively digital PCR was more sensitive than ARMS in detecting T790M mutation both in pre- and post-TKI plasma samples 31.1% *vs* 5.5% (*P* < 0.001) and 43.0% *vs* 25.2% (*P* = 0.001), respectively [90]. Quantitative dynamic monitoring of T790M mutation by digital PCR is useful to predicted the clinical outcomes of EGFR TKIs using plasma ctDNA, as serial re-biopsies using tissue is practically impossible [89-92]. In detecting T790M mutation ddPCR has high sensitivity and specificity compared to cobas, BEAMing, ARMS and conventional PCR [70, 85, 93].

Targeted NGS using Ion Torrent Personal Genome Machine detected T790M resistant mutation in 60% of the cases which were not diagnosed by other conventional platforms. In addition to EGFR mutations other oncogenic mutations were detected which may play a role in TKIs resistance. This high throughput analysis of NGS elucidates the importance of such analysis in targeted therapy [94]. Two other studies also demonstrated the use of targeted NGS in detection of resistant mutations both in tissue and plasma even at low abundance rate [95, 96]. Mass spectrometry (MALDI-TOF-MS) compared to direct sequencing yielded good results with detection rates of 83.3 and 33.3% respectively for T790M mutation in tissue. The results of MALDI-TOF-MS showed good correlation with NGS [97].

Cobas is a semi-quantitative method used frequently in mutational analysis using tissue or plasma [21, 70, 87, 98-101]. Thress *et al.* reported concordance of 78.6% between tumor tissue and plasma using this method, another study indicated a positive percentage agreement of 64% between tissue and plasma [70, 98]. Quantification of T790M mutation using cobas and NGS significant correlation between the two tests (*P* < 0.001) with concordance rate of 95%. The sensitivity and specificity of cobas and NGS was 72% and 100% to that of 74% and 100%, respectively. Though PCR based techniques can identify only the known mutations, they are preferred over NGS due to the advantages attributed to their ease, turnaround time and cost [100].

## PREVALENCE OF T790M IN PRE-TKI AND POST-TKI NSCLC PATIENTS

Ethnic variations are observed in EGFR mutations. The mutation rate among east Asians is 30-40% among east Asians when compared to 5-13% in Caucasians, signifying the importance of molecular analysis in east Asian popluations [102]. Among the EGFR mutations, the T790M mutation occurs in less than 5% of the untreated EGFR mutated tumors and occurs to about 50% of the EGFR mutated tumors that acquire resistance to the first generation TKIs [12, 77, 103]. Tables 3 and 4 summarise the prevalence of T790M mutations in pre- and post-TKI NSCLC patients using tissue and plasma samples. Though for most of the studies patients ethnic details are not reported. Dividing all the studies into Asian and non-Asian. Asians studies have used cobas, ddPCR, BEAMing, ARMS, direct sequencing and Ion torrent PGM platforms for detecting T790M mutation [21, 70,71, 75, 81, 84, 87, 88, 94, 101, 104, 105]. The frequency of T790M mutation ranged from 0- 35% with most of the studies reporting less than 5% before TKI administration in NSCLC [71, 75-77, 79, 84, 86-88, 99, 100, 106-108]. Three studies reported more than 50% of T790M mutation in patients before TKI [86, 92, 105]. This high frequency could be attributed to small sample size in one of the studies [92] and to high sensitivity of the detection methods (ddPCR and colony hybridisation) used to detect low abundance T790M mutation in the other two studies [86, 105]. The incidence of T790M mutation in after TKI ranged from 0-70% with most studies reporting around 50% of this resistant mutation in NSCLC patients [21, 74, 75, 78, 80, 81, 84, 85, 87, 88, 94, 101, 104, 106, 109-111]. This low detection rates in few of the post-TKI studies of T790M mutation rate could be attributed to the technology used for detection (direct sequencing) and heterogeneity of the tissue sample [77, 81].

Non-Asian studies used ARMS, cobas and PNA-PCR for mutation detection [26, 99, 107, 111]. The incidence of post-TKI T790M around 50-60% [99, 110, 111]. The percentage of T790M mutation directly correlates with the treatment duration of the first and second line TKI for acquiring resistance to these TKIs. The variation in the rate may also be attributed to the differences in sensitivities of the testing platforms. The rate of T790M detected in tissue and plasma also varies as evident from the various studies (Table 3 and Table 4). Moreover, Sueoka-Aragane et al. demonstrated that T790M mutation was frequently detected in certain subgroups of patients like smokers, males, in patients with exon 19 deletion and in patients with new lesions [112].

Several studies demonstrated the prevalence of T790M in Chinese populations using various technologies. Zhao et al validated three platforms RTD-PCR sequencing, TaqMan probe PCR and Sequenom MassArray for specific detection of EGFR T790M mutation and found that all three platforms detected T790M in seven cases from 78 tissue samples [113]. The ddPCR showed better sensitivity and specificity over qPCR in detecting EGFR mutations in tissue samples and it detected T790M mutation (6.4%) which were missed by qPCR in pre-TKI patients [93]. ARMS detected T790M mutation in 48.3% in post-TKI patients whereas no mutation was detected in pre-TKI Chinese NSCLC patients [84]. Another study reported T790M mutation in 36.1% in TKI resistant patients using NGS [114].

## CONCLUSION

In this review, we compared various companion diagnostic platforms for EGFR T790M testing. Multiple platforms like cobas, BEAMing, ddPCR and NGS are capable of detecting EGFR TKI resistant mutations in NSCLC patients though they differ in their sensitivity, specificity and turnaround time. In cases that demand quantification of mutation BEAMing, ddPCR and NGS could take a lead. More prospective studies to monitor the EGFR T790M in plasma ctDNA during or after EGFR TKI treatment are warranted.

Overall the data suggests that plasma testing is useful compared to tissue especially in patients with EGFR T790M resistant mutations where continuous monitoring is mandate. Other bodily fluids can also be investigated as potential alternatives in real-time monitoring for targeted therapy in EGFR mutated NSCLCs.
